# Comparative Analysis of the Gut Microbiota of Mandarin Fish (*Siniperca chuatsi*) Feeding on Compound Diets and Live Baits

**DOI:** 10.3389/fgene.2022.797420

**Published:** 2022-05-18

**Authors:** Xiao Chen, Chengfei Sun, Junjian Dong, Wuhui Li, Yuanyuan Tian, Jie Hu, Xing Ye

**Affiliations:** ^1^ Key Laboratory of Tropical and Subtropical Fisheries Resource Application and Cultivation, Ministry of Agriculture and Rural Affairs, Pearl River Fisheries Institute, Chinese Academy of Fishery Sciences, Guangzhou, China; ^2^ Key Laboratory of Exploration and Utilization of Aquatic Genetic Resources, Ministry of Education, Shanghai Ocean University, Shanghai, China; ^3^ Key Laboratory of Freshwater Aquatic Genetic Resources, Ministry of Agriculture, Shanghai Ocean University, Shanghai, China

**Keywords:** *Siniperca chuatsi*, compound diet, bait, gut microbial communities, 16SrRNA sequencing, growth rate

## Abstract

*Siniperca chuatsi* feeds on live fry throughout their life. The sustainable development of its farming industry has urgently necessitated the development of artificial diets to substitute live baits. It has been demonstrated that gut microbiota assists in feed adaptation and improves the feed conversion rate in fish. Therefore, this study aimed to understand the potential role of intestinal microorganisms in the domestication of *S. chuatsi* with a compound diet. Accordingly, we performed 16S rRNA sequencing of the gut microbial communities in *S. chuatsi* groups that were fed a compound diet (including large and small individuals) and live baits. A total of 2,471 OTUs were identified, and the large individual group possessed the highest number of unique OTUs. The α-diversity index of the gut microbiota in groups that were fed a compound diet was significantly higher (*p* < 0.05) than that in the live bait group. There were no significant differences in the α-diversity between the large and small individual groups. However, relatively higher numbers of *Lactococcus*, *Klebsiella*, and *Woeseia* were observed in the intestines of the large individual group. Prediction of the metabolic function of the microbiota among these three fish groups by Tax4Fun revealed that most metabolic pathways, such as glycan metabolism and amino acid metabolism, were typically more enriched for the larger individuals. The results indicated that certain taxa mentioned above exist in large individuals and may be closely related to the digestion and absorption of compound diets. The present study provides a basis for understanding the utilization mechanism of artificial feed by *S. chuatsi*.

## Introduction

The intestinal microbiota of fish participates in the assimilation of food substances, affecting the nutrition, growth, reproduction, general population dynamics, and health status of the host (Bereded et al., 2021). Studies on various fish species, such as *Danio rerio*, *Oreochromis niloticus,* and *Salmo salar*, have demonstrated that the gut microbiome plays a key role in host development. The intestinal microbial community can boost digestibility, improve the intestinal microbial balance, and protect the host from intrusive pathogens by having advantageous impacts on feedstuff absorption. For instance, intestinal microbiomes can produce a variety of digestive enzymes, such as amylase, protease, and alkaline phosphatase, that facilitate feed digestion in fish (Burr et al., 2005; Suzer et al., 2008; Dimitroglou et al., 2010; Mirghaed et al., 2018; Ringøb et al., 2020; Zhu et al., 2021). Some intestinal flora species improve the digestive efficiency of artificial feeds in fish by correcting inter-microbial interactions or by influencing fish growth and metabolism through positive effects on growth-related genes (Zuenko et al., 2017; Zhang et al., 2018). Dvergedal et al. (2020) deemed that the intestinal microbiota may have a direct impact on distal intestine biomolecules or an indirect association with fish metabolism for host growth (Dvergedal et al., 2020). Furthermore, the health of the villi is one of the crucial factors that affects nutrient absorption. Won et al. (2020) hypothesized that intestinal flora indirectly promotes feed utilization in fish by altering intestinal histology (Won et al., 2020).

*Siniperca chuatsi*, commonly known as mandarin fish (order Perciformes, family Serranidae, and genus *Siniperca*), is mainly distributed in the rivers of East Asian countries, including China, Vietnam, and Korea. It is an aggressive benthic fish that feeds on live fry throughout its life. *S. chuatsi* was first successfully bred in captivity in China in the 1970s and has since become an important freshwater aquaculture species in the country (Sun et al., 2017). However, feeding *S. chuatsi* with live bait fish poses many challenges, including ensuring the supply according to the size and demand of *S. chuatsi* during the cultural process. An insufficient size or shortage of the bait fish would lead to the mortality of mandarin fish in just a few days. Additionally, bait fish may carry pathogens, such as *Siniperca chuatsi* rhabdovirus and infectious spleen and kidney necrosis virus (Guo et al., 2017), and increase the risk of infection in the cultured *S. chuatsi*. Therefore, the adaptation of feedstuff, instead of the use of live bait, has emerged as a potential strategy that is crucial for the healthy and sustainable development of *S. chuatsi* farming. Recently, there have been attempts to domesticate *S. chuatsi* with an artificial diet, but the uneven size and low survival rates of *S. chuatsi* often occur during the process of domestication. We conjectured that these issues were related to fish gut microbial communities. In addition, we also hypothesized that there would be differences in the gut microbial communities between fish fed with live baits vs. those fed a compound diet. However, little is known about the long-term (months) change in gut microbial communities in feedstuff adaptation of *S. chuatsi*.

Research on the adaptation to compound feed by *S. chuatsi* has indicated that adaptation is related to intestinal microorganisms. Sun, (2016) found that there were tractable and non-tractable individuals in the wild *S. chuatsi* population, and there were significant differences between the gut microbiota of these individuals (Sun, 2016). Previous studies investigating the gut microorganisms of *S. chuatsi* all studied them at the fry stage (weighing approximately 50 g), whereas there are no comparable studies that investigate the gut microbiome of adult *S. chuatsi* after one breeding cycle of feeding on a compound feed.

In the present study, to understand the effects of feedstuff vs. live bait feeding on the gut microbiota of adult *S. chuatsi*, high-throughput sequencing technology was employed to sequence the 16S rRNA V3+V4 region of gut microorganisms of adult *S. chuatsi* (large and small individuals) fed with a compound diet vs. live bait fish. The structural composition of the gut microbiome was classified and compared. This study therefore lays the foundation for understanding the utilization mechanism of artificial feed by *S. chuatsi*.

## Materials and Methods

### Ethical Statement

All experiments were carried out in accordance with the “Guidelines for the Protection and Use of Laboratory Animals in China.” All experimental procedures and sample collection were approved by the Animal Experimentation Ethics Committee of the Pearl River Fisheries Research, Chinese Academy of Fishery Sciences.

### Sampling

*S. chuatsi* fed on a compound diet was provided by the Longjiang culture base in Shunde, Guangdong Province. Completely domesticated *S. chuatsi* fry (12 cm) had been placed in net cages and fed with a compound feed throughout the experiment (Supplementary Table S1), with the particle size of the artificial feed adjusted following the growth stage of the fish. Six large (795.67 g ± 40.64) and six small individuals (338.00 g ± 11.36) were selected. *S. chuatsi* feeds on live baits were collected from the farming ponds of Yushun Agriculture and Fishery Technology Service Co., Ltd. in Guangdong Province. The size of live bait is adjusted according to the growth stage of *S. chuatsi* and the total length of the live bait does not exceed 40–60% of the total length of *S. chuatsi*. Six *S. chuatsi* fed live baits were randomly selected (439.83 g ± 135.16) (Table 1). Each group of *S. chuatsi* was anesthetized using MS-222 and dissected. Their intestinal contents were removed and collected in 1.5 ml centrifuge tubes, placed immediately in liquid nitrogen for flash freezing, and stored in a −80°C refrigerator for high-throughput sequencing. Six large individuals feeding on the compound diets were named as Feed-large (Fl); Six small individuals feeding on the fish feedstuff were recorded as Feed-small (Fs); Six *S. chuatsi* feeding on baits were recorded as Bait (B). The water samples from the compound feed groups and the live bait group were recorded as Water-Feed (WF) and Water-Bait (WB) groups, respectively. The samples consisted of 18 *S. chuatsi* gut samples and six water samples, making a total of 24 samples.

**TABLE 1:** Body weights of adult large individuals (Fl), small individuals (Fs) and bait groups (B) of S. chuatsi.

Number	Culture-cycle	Weight	Feed
Fl1	9 months	767	Compound diet
Fl2		806	
Fl3		822	
Fl4		719	
Fl5		835	
Fl6		825	
Fs1		349	
Fs2		345	
Fs3		316	
Fs4		336	
Fs5		334	
Fs6		348	
B1	7 months	246	Live *Cirrhinus molitorella* fry
B2		538	
B3		537	
B4		563	
B5		255	
B6		500	

### DNA Extraction

DNA extraction was performed immediately after the gut microbiota samples were retrieved from the −80°C refrigerator. The Cetyltrimethyl ammonium bromide (CTAB) method is a commonly used method for the extraction of gut microbial DNA; it can disrupt the membrane structure, deform the protein, and increase the solubility of DNA in the extraction solution. The standardized operating procedure of the CTAB method was used to extract genomic DNA from the gut microbiota of *S. chuatsi* (Wilson, 2001). The extracted DNA was detected for purity using 1% agarose gel electrophoresis. The microflora in the water samples was obtained by vacuum filtration of the water samples, which were then subjected to high-throughput sequencing (Novogene, Beijing, China).

### 16S rRNA Amplification and Sequencing

In this experiment, 341F (341F: 5′-CCTAYGGGRBGCASCAG-3′) and 806R (806R: 5′ GGACTACNNGGGTATCTAAT-3′) were used as primers to amplify the high variation region of V3-V4 in the 16S rRNA gene of the microorganisms from each sample group (Berg et al., 2012). Polymerase Chain Reaction (PCR) mixtures contained 15 µl of Phusion^®^ High-Fidelity PCR Master Mix (New England Biolabs), 0.2 µM of each primer and 10 ng target DNA, and cycling conditions consisted of a first denaturation step at 98°C for 1 min, followed by 30 cycles at 98°C (10 s), 50°C (30 s) and 72°C (30 s) and a final 5 min extension at 72°C, and the PCR products were examined with 2% agarose gel electrophoresis and purified by 1×TAE agarose gel electrophoresis with 2% concentration. The gel products were purified and recovered using Qiagen Gel Extraction Kit (Qiagen, Germany).

Sequencing libraries were constructed using TruSeq DNA PCR-Free Sample Preparation kit (Illumina, United States) following manufacturer’s recommendations and index codes were added. The library quality was assessed on the Qubit@ 2.0 Fluorometer (Thermo Scientific, United States) and Agilent Bioanalyzer 2,100 system (Shanghai, China). At last, the library was sequenced on an Illumina NovaSeq platform and 250 bp paired-end reads were generated.

### Data Analysis

All the raw data have been deposited in the NCBI Sequence Read Archive database (SRA accession number is PRJNA795654). Paired-end reads was assigned to samples based on their unique barcode and truncated by cutting off the barcode and primer sequence. Paired-end reads were merged using FLASH (VI.2.7), which was designed to merge paired-end reads when at least some of the reads overlap the read generated from the opposite end of the same DNA fragment, and the splicing sequences were called raw tags (Magoc and Salzberg, 2011). Quality filtering on the raw tags were performed to obtain the high-quality clean tag according to the QIIME (V1.9.1) quality controlled process (Caporaso et al., 2010). The tags were compared with Silva database (https://www.arb-silva.de/) using UCHIME algorithm to detect and remove chimera sequences (Edgar et al., 2011). Sequences with 97% similarity were grouped into the same Operational Taxonomic Units (OTUs). Representative sequence for each OTU was screened, the Silva Database was used based on Mothur algorithm to annotate taxonomic information (Quast et al., 2013). In order to study phylogenetic relationship of different OTUs and the difference of the dominant species in different samples (groups), multiple sequence alignment were conducted using the MUSCLE software (Version 3.8.31).

The α-diversity index was calculated for each group of samples by using QIIME (v. 1.9.1) software. Microbiota abundance was evaluated using Chao1 and ACE values, and microbiota diversity was evaluated by using Simpson’s index. *p*-value was determined using SPSS 21.0 (SPSS, United States) software. The UniFrac distance matrix was used for the analysis of *β*-diversity, and the R package (Version 2.15.3) was used to perform principal coordinate analysis (PCoA) and generate bar graphs, Venn diagrams, and heat maps. The unweighted UniFrac distances were used to assess the degree of variation in gut microbe between individuals. Using Tax4Fun software, the OTU information of 16S was used to predict the metabolic function of the microbiota based on the linear relationship between the SILVA database and the prokaryotic classification in the Kyoto Encyclopedia of Genes and Genomes (KEGG) database (Aßhauer et al., 2015). Wilcoxon test and *t*-test were applied to assess divergences in the gut microbes among groups. *p*-value ＜0.05 was considered statistically significant.

## Results

### Sequencing Results

**FIGURE 1:**
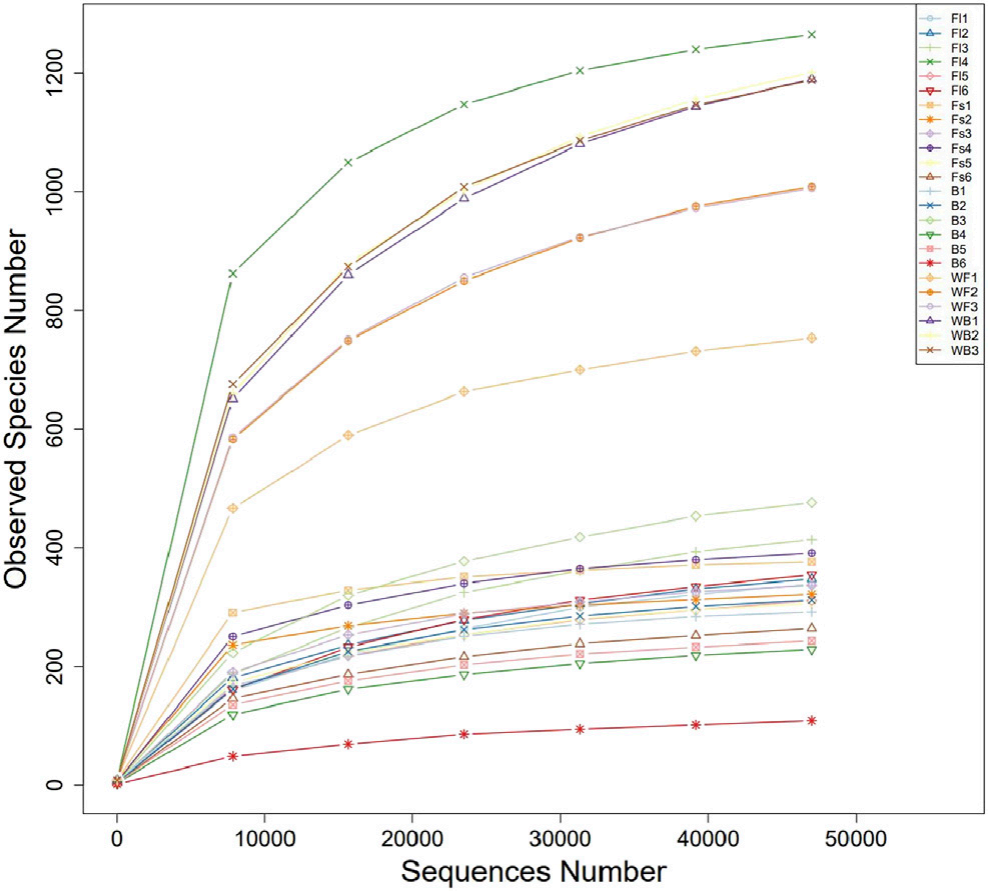
Rarefaction curves of microbial communities sampled from the gastrointestinal tract of *Siniperca chuatsi*.

To identify the microbial communities in the gut microbiota of *S. chuatsi* fed with either artificial feed or live baits, a 16S rRNA high-throughput sequencing study was performed. Subsequent data analysis revealed that a total of 1,517,928 effective sequences were obtained from 24 samples. Furthermore, these sequences were clustered into 5,699 OTUs including 79 phyla, 169 classes, 320 orders, 449 families, and 697 genera (Table 2). Additionally, the coverage index of the rarefaction curve of each sample reached a saturation plateau with high sequencing quality and coverage, thereby reflecting that the sequencing data were adequate to provide consistent and unbiased estimates of species richness (Figure 1).

**TABLE 2:** Effective Tags, abundance, and alpha diversity index of the gut microbiota in each Siniperca chuatsi group. ACE: estimated number of OTUs contained in the microbiota; Chao1: estimated total number of species contained in the microbiota samples; Simpson’s index: characterize the diversity and homogeneity of species distribution within the microbiota, this study used Simpson’s index of diversity (1–D).

Group	Total effective Tags	OTUs	ACE	Chao1	Simpson
Fl	387,396	1,260	440.04 ± 50.35^a^	416.03 ± 52.53^a^	0.70 ± 0.05^a^
Fs	365,122	708	362.89 ± 35.60^a^ ^,^ ^b^	373.57 ± 29.40^a^	0.82 ± 0.09^a^
B	387,509	503	313.38 ± 30.87^b^	305.55 ± 30.80^b^	0.54 ± 0.09^b^
WF	185,900	1,411	926.07 ± 116.18^a^	913.12 ± 116.36^a^	0.97 ± 0.01^a^
WB	192,001	1,817	1196.81 ± 4.12^a^	1178.72 ± 10.55^a^	0.97 ± 0.01^a^

a *p* > 0.05, no significant difference

b *p* < 0.05, significant difference.

Fl and Fs, large individuals and small individuals feeding on the compound diets, respectively; B, S. chuatsi feeding on baits; WF, the water samples from the compound feed group; WB, the water samples from the bait group (same as below).

### Analysis of Microbiome Diversity in the Gut and the Waterbodies of Each *S. chuatsi* Groups

**FIGURE 2:**
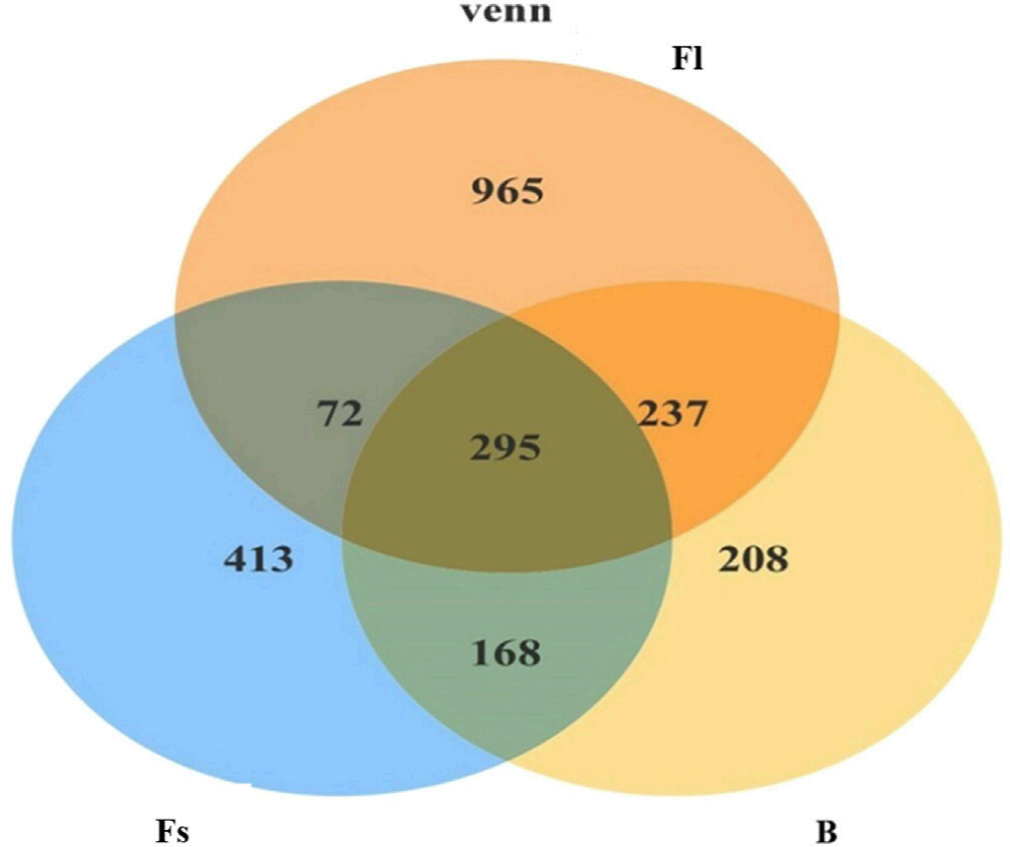
Venn diagram comparing the observed OTU of intestinal bacterial communities from shared and unique to each *S. chuatsi* group.

The analysis of unique or shared OTUs of small- (Fs) and large-sized (Fl) *S. chuatsi* administered artificial feed or adult *S. chuatsi* fed on live baits (B) demonstrated that 295 OTUs were common to the three groups. The number of OTUs unique to the Fl, Fs, and B groups was 1,260, 708, and 503, respectively (*p* < 0.05). Furthermore, the number of OTUs was the highest in group Fl (Figure 2). The α-diversity metrics, namely the abundance of species within a community and the number and distribution of each species, include ACE values, Chao1 values, and Simpson’s index of diversity (1–D). The result illustrated that α-diversity indices were significantly different between the groups fed artificial diets and the live baits (*p* < 0.05). However, there were no significant differences in the α-diversity index of gut microorganisms between group Fl and group Fs (*p* > 0.05). The divergences in α-diversity indicators between the WF and WB groups of the two water samples were not significant (*p* > 0.05) (Table 2).

### Share and Differences in the Microbiota Composition in the Gut and Waterbodies of Each *S. chuatsi* Group

Analysis of the structure of the gut microbiome of each *S. chuatsi* experimental group revealed that the composition of the dominant microbial communities were similar in all the groups. At the phylum level, the gut microbiota of three groups *S. chuatsi* were dominated mainly by Proteobacteria followed by Fusobacteria and Firmicutes, which together accounted for 86.55%–95.23% of the total population, the relative abundance was not considerably different (*p* > 0.05). Furthermore, the proportions of each category were diverse in each group, the relative abundance of Firmicutes was the highest in group Fl while that of Bacteroidetes and Actinobacteria were the richest in group Fs. Meanwhile, the relative abundance of Proteobacteria and Cyanobacteria were the most abundant in group B (Table 3). Remarkably, no significant differences (*p* > 0.05) were detected in the microbiota structure between the water samples of the two culture environments corresponding to the groups fed with the compound diet and live baits. Furthermore, the dominant microbiota in both the waterbodies were Proteobacteria, Actinobacteria, Bacteroidota, Cyanobacteria, Verrucomicrobiota, Firmicutes, Chloroflexi, Kapabacteria, and Fusobacteriota (Table 3).

**TABLE 3:** Relative abundance of microflora at the phylum level in the gut samples and water environment of each S. chuatsi group.

The relative abundance of the microbial community in the intestinal tract of *S.chuatsi* and water (%)					
Group	Fl	Fs	B	WF	WB
phylum					
Proteobacteria	57.81	49.28	69.45	52.36	52.28
Fusobacteriota	24.17	27.58	18.7	1.71	0.38
Firmicutes	13.25	9.77	5.26	3.14	1.22
Bacteroidota	0.74	6.17	0.42	7.98	8.05
Actinobacteriota	0.38	2.21	2.44	11.68	14.94
Cyanobacteria	0.3	0.28	1.42	3.2	4.46
Verrucomicrobiota	0.36	0.43	0.39	3.14	2.18
Chloroflexi	0	0	0	2.72	0.42
kapabacteria	0	0	0	1.79	1.28

At the genus level, the relative abundance of *Lactococcus*, *Klebsiella*, *Escherichia-Shigella*, *Woeseia*, and *Bacteroides* was significantly higher in the gut of *S. chuatsi* was fed with a compound diet than in group B. The relative abundance of *Lactococcus*, *Woeseia*, *and Klebsiella* was the highest in group Fl. Moreover, the relative abundance of *Escherichia-Shigella* and *Bacteroides* was significantly richer in group Fs than other groups (Table 4).

**TABLE 4:** Microbial with differences in abundance at the genus level between groups (%).

Genus	Fl	Fs	B
*Lactococcus*	8.32	0.18	0.28
*Klebsiella*	3.3	0.09	0
*Escherichia shigella*	0.21	2.51	0.21
*Bacteroids*	0.26	2.54	0.11
*Woeseia*	1.64	0	0.09

**FIGURE 3:**
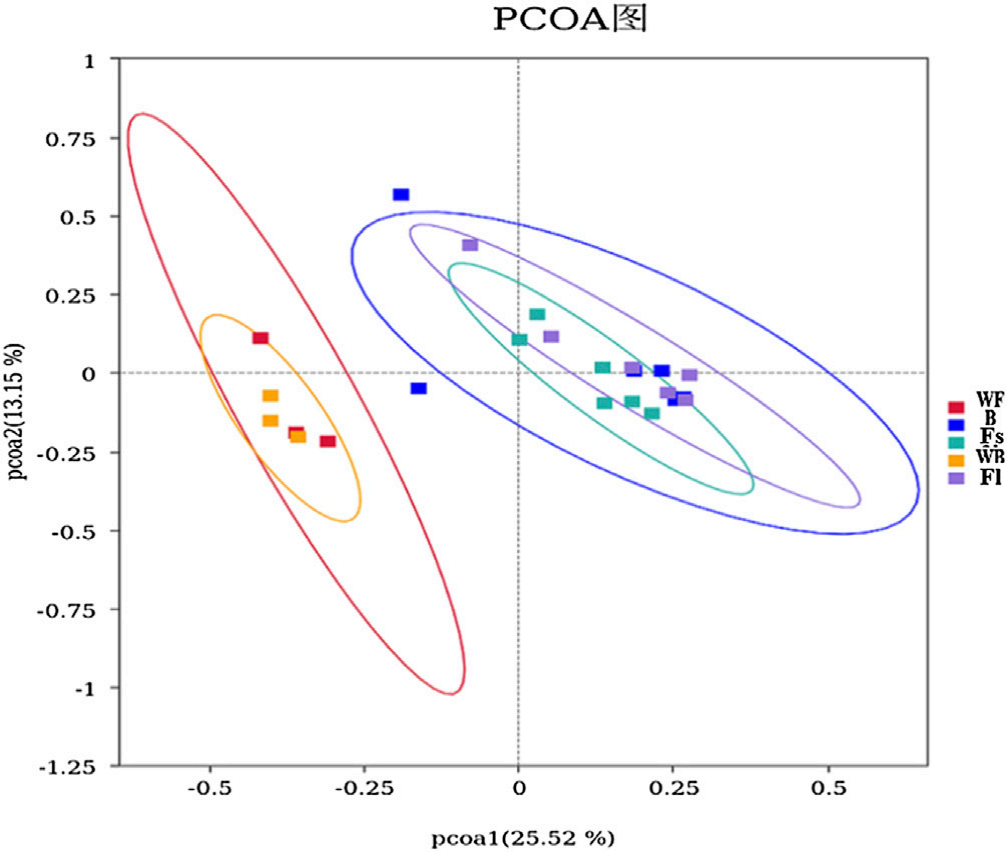
PCoA analysis of microorganisms in the gut and water environment of each *S. chuatsi* group based on unweighted-UniFrac. Individual samples from different *S. chuatsi* groups and aquatic environments are shown as squares of different colors in the coordinate system, with similar samples showing a clustering trend.

The *β*-diversity analysis involves a comparative analysis of the microbiota composition of the different samples. Moreover, the PCoA was performed on all samples by using unweighted-UniFrac distances, and the results revealed that experimental fish (compound feeds and prey fish groups) and the two water samples were clearly divided into two groups, indicating that the microbiota in the experimental fish and the cultural water was significantly different, whereas group Fl and group B were clustered into one group indicating that their gut microbiota composition was similar (Figure 3). Nevertheless, Adonis indicated that the microbial community of the feed groups was not significantly different from the bait group (R2 = 0.0526, *p* = 0.541).

### Identification of Differential Gut Microbiota Between Different *S. chuatsi* Groups

The top 35 species of gut microbiota in the groups feeding on compound diets and live baits were compared with those in their respective waterbodies to obtain the microorganisms that were unique to the gut of the experimental fish. The results displayed that some microbes, such as *Klebsiella aerogenes*, *Lactococcus lactis*, and *Bacteroides plebeius*, were unique to the formulate feed groups; whereas *Acinetobacter sp CIP 53.82*, *Rhodococcus fascians*, and *Acinetobacter lwoffii*, etc., were specific to group B.

The top 35 species of the gut microbiota of group Fl and group Fs were then compared with those of their respective waterbodies. *Pseudomonas balearica*, *Sphingobacterium faecium*, *Lactococcus lactis* and *Roseomonas ludipueritiae* were discovered to be unique to the group Fl. The relative abundance of *Klebsiella aerogenes*, *Kosakonia cowanii*, and *Lactococcus garvieae* was significantly richer in group Fl than in group Fs, whereas *Bacteroides plebeius*, *Anaerotignum lactatifermentans*, and *Bacteroides ovatus* were unique to group Fs. The abundance of *Stenotrophomonas maltophilia*, *Escherichia coli*, and *Bacteroides dorei* was significantly higher in group Fs than in group Fl (Supplementary Table S2).

### Predictive Analysis of Microbiota Function With Tax4Fun

**FIGURE 4:**
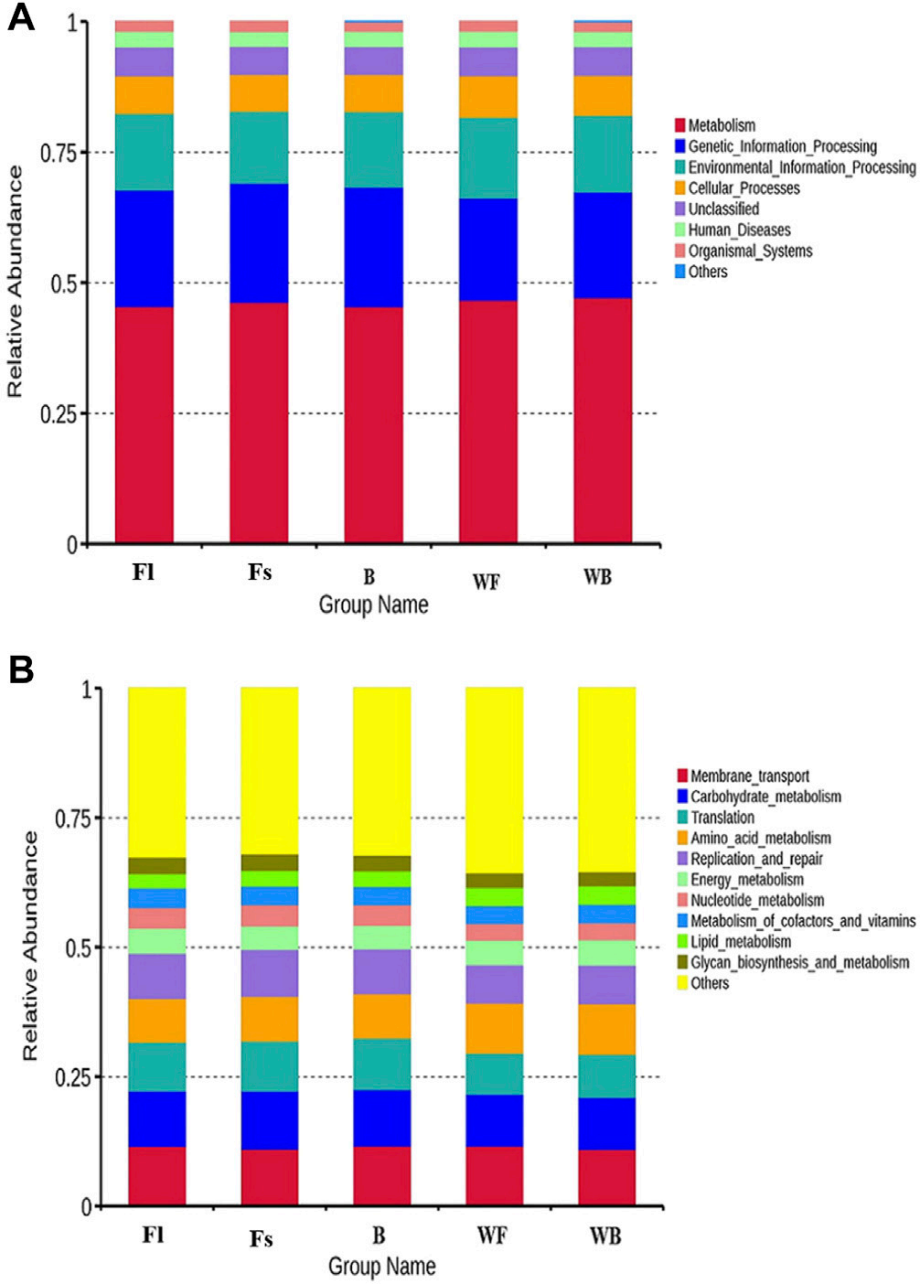
Top 10 metabolic pathway sub-functions of the KEGG signaling pathway at the level 1 **(A)** and level 2 **(B)**.

To perform a functional analysis of gut microbiota associated with *S. chuatsi* groups fed with either an artificial feed or live baits, the Tax4Fun was applied. The analysis revealed a total of eight metabolic pathways (including unclassified and others) were annotated at level 1 of the KEGG metabolic pathway in the three groups of *S. chuatsi* gut microorganisms, of which the OTUs involved in metabolism were the most abundant, followed by OTUs involved in genetic information process and environmental information processes. Besides, a total of 44 pathways were annotated at level 2 of the KEGG metabolic pathway, with membrane transport, glycan metabolism, translation, amino acid metabolism, and replication and repair being the five most enriched pathways (Figures 4A,B).

**FIGURE 5:**
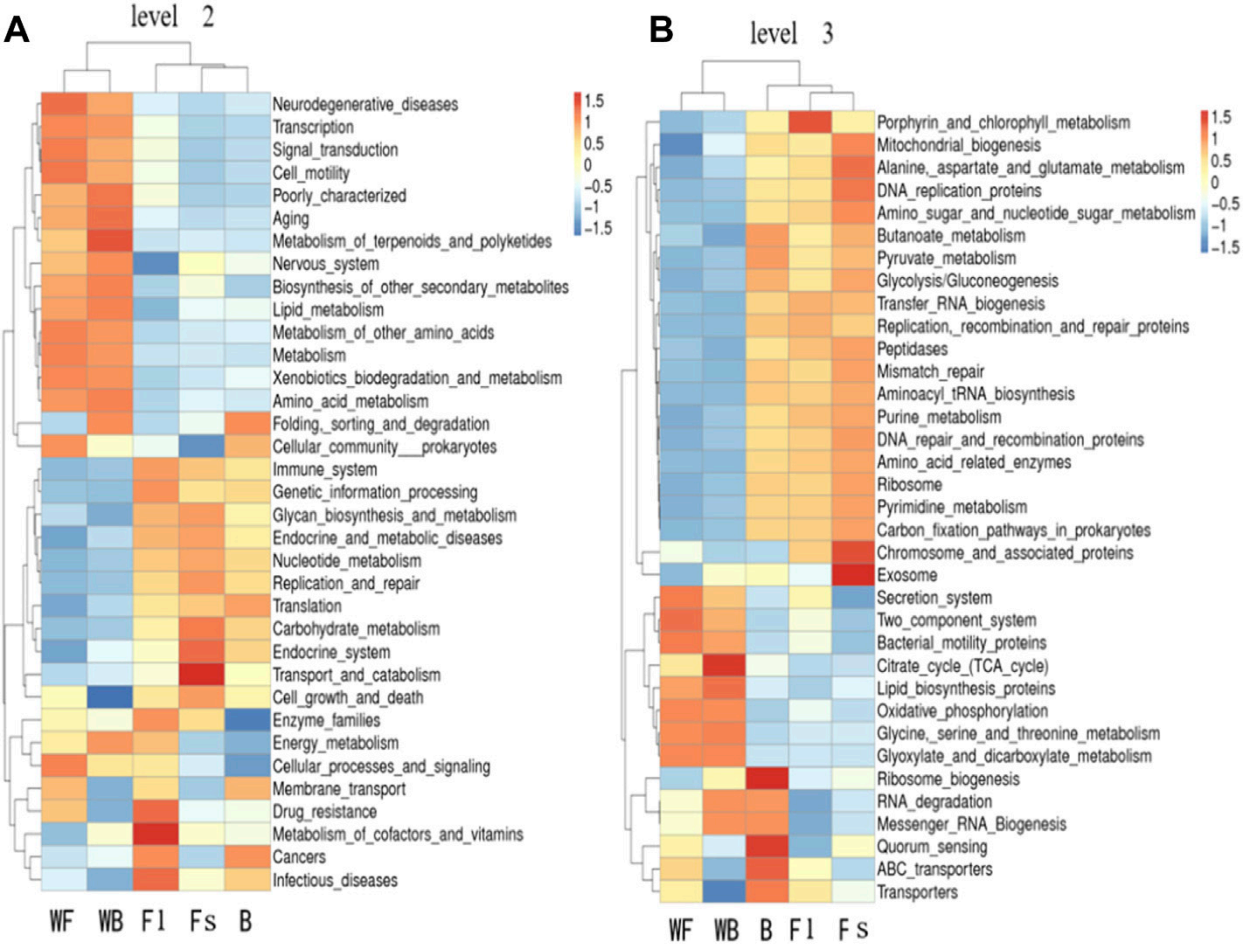
Tax4Fun functional annotation clustering heat map at the level 2 **(A)** and level 3 **(B)**. The color of the bars indicates the relative abundance of microorganisms in each function for each group of samples; red and blue indicate the amount of that function present in the sample, respectively.

The predictive and clustering analysis of the metabolic functions of the three groups of *S. chuatsi* gut microorganisms revealed that the abundance associated with metabolic sub-pathways, such as metabolism of cofactors and vitamins, energy metabolism, cellular processes and signaling, and enzyme families, were significantly more abundant in group Fl than in group B. However, those associated with cellular processes and signaling, membrane transport, and energy metabolism were more abundant in group Fl than in group Fs. Further analysis revealed that microorganisms associated with porphyrin and chlorophyll metabolism, transfer RNA biogenesis and replication, as well as recombination and repair proteins, were significantly more abundant in group Fl than in group B. However, those associated with the secretion system, transporters, ABC transporters, replication, recombination and repair proteins were more abundant in group Fl than in group Fs (Figure 5).

We used the Tax4Fun to predict the pathways closely related to the feed adaptation and growth of *S. chuatsi*, founding that the gut microbial communities of *S. chuatsi* with different feeds and growth rates exhibited different functional diversities. KEGG pathway further analysis revealed that the relative abundance of the OTUs mainly involved in carbohydrate metabolism and enzyme activity was higher in the group Fl, indicating that the growth performance of *S. chuatsi* in this group was directly influenced by the carbohydrate metabolism capability and intestinal environment equilibrium. However, the relationship between the metabolic regulation of intestinal microbiota and the changes in *S. chuatsi* needs to be further investigated.

## Discussion

To the best of our knowledge, the present study is the first of its kind to investigate the gut microbial population of *S. chuatsi* cultivated over months. The intestinal microbiota of groups fed with compound feed (containing groups of large and small growth, group Fl and group Fs) and live bait for months were studied using high-throughput sequencing techniques. The results showed that there are common core microbiota that exist among the groups investigated and that the composition and diversity of gut bacteria varied considerably across the artificial diet and live bait groups. Moreover, we report the presence of the genus *Woeseia*, for the first time, in the intestines of fish.

### Core Microbiota Shared by Mandarin Fish Feeding on Compound Feedstuff and Live Bait

In this study, 79 bacterial phyla and 295 shared OTUs were detected in the intestinal microbiota of three *S. chuatsi* groups. The OTUs of the intestinal microbiota were higher in the groups fed on compound diets (Fl and Fs groups) than those in group B. Nevertheless, the dominant bacteria in the intestinal tract of *S. chuatsi* fed with either compound diets or baits were mainly Proteobacteria, Fusobacteria, and Firmicutes. Bacteria from these three phyla have also been documented to be dominant in the gut microbiota of other fishes, such as rainbow trout (*Oncorhynchus mykiss*), carp (*Cyprinus carpio*), and crucian carp (*Carassius auratus*), suggesting that these three bacterial phyla are common colonizers of fish guts and that they may play important biological roles in fishes (Eichmiller et al., 2016; Parshukov et al., 2019; Zou et al., 2020; Bereded et al., 2021).

The phylum Proteobacteria is the most extensive in the gut of aquatic organisms and is widely involved in the nutrient metabolism of fish (Kersters et al., 2006). Certain Proteobacteria in the gut of healthy fish may significantly contribute to digestive function by secreting enzymes associated with environmental adaptation, which can enhance the digestion of food by the host, while other enzymes are involved in riboflavin and biotin synthesis pathways (Magnúsdóttir et al., 2015; Xu et al., 2019; Liu and Peng, 2021). Fusobacteria are mostly obligate anaerobic rod-shaped bacteria that can ferment different substrates (carbohydrates or amino acids) into various organic acids (Zhu et al., 2020). They regulate the intestinal pH and enzyme activity or control conditioned pathogens in the intestine to regulate probiotics and improve nutrient digestibility and feed utilization. Some members have been reported to participate in vitamin B12 metabolism, while a few studies have demonstrated a relationship between the content of animal proteins in feed and the relative abundance of Fusobacteria (Michl et al., 2017; Busti et al., 2020; Zhang et al., 2021). Firmicutes are associated with carbohydrate metabolism and absorption in fish and affect fatty acid adsorption and lipid metabolism. They produce a variety of digestive enzymes to break down nutrients in different baits, stimulate host metabolism, and increase fatty acid bioavailability by altering bile salt production or composition (Semova et al., 2012). Firmicutes, such as Ruminococcaceae, Lachnospiraceae, and Clostridiaceae, can produce butyrate, which is the most vital short-chain fatty acid for fish and is beneficial for fish intestinal health (Smriga et al., 2010; Nuriel-Ohayon et al., 2016; Esquivel-Elizondo et al., 2017; Zhang et al., 2021).

### Feeding on Compound Feeds Altered the Intestinal Microbiota Composition of *S. chuatsi*

In the present study, we discovered that particular taxa may be useful in predicting growth rate, with the fast-growing taxa seeming to have a mutually beneficial interaction with the host, including *Lactococcus*, *Klebsiella*, and *Woeseia*.

*Lactococcus*, belonging to the phylum Firmicutes, is considered the most effective antibiotic alternative and has been used extensively in aquaculture (Zorriehzahra et al., 2016; Ringøa et al., 2018). Multiple previous studies have already verified that some bacteria of *Lactococcus* spp. may significantly contribute to fish growth (Chapagain et al., 2019; Zhang et al., 2020). They have been documented to affect the palatability of the feed and alter the intestinal microbiome and the morphology of the microvilli on the intestines, thereby making the fish more receptive to feed and increasing their food intake (Dawood et al., 2016; Xia et al., 2018; Zhu et al., 2020). Additionally, *Lactococcus* sprayed into the basal diet can effectively improve the growth rate of *Astacus leptodactylus* by stimulating the activity of digestive enzymes, such as lipase, amylase, alkaline phosphatase, and protease, in the intestinal tract (Valipour et al., 2019). *Lactococcus* was the most abundant in group Fl of mandarin fish, with a high abundance of 8.32%. We hypothesized that this bacterium indirectly facilitates the digestion and absorption of the compound feed in mandarin fish, hence boosting individual development.

*Klebsiella*, including *K. aerogenes*, *K. pneumoniae*, and *K. ozaenae*, is an important conditioned pathogen in humans and animals (Lee et al., 2021). Further projected annotations, however, have indicated that *K. aerogenes* is the most prevalent species in the *Klebsiella* genus. *K. aerogenes* is a common facultative anaerobic digestive bacterium that can degrade essentially all amino acids, except for the three branched chain amino acids leucine, valine, and isoleucine (Reitzer, 2005). *K. aerogenes* activates proline oxidase in the presence of large amounts of protein to utilize the proline, and in mixed cultures with *Escherichia coli* it leads to the Stickland reaction. The Stickland reaction is the simplest method of amino acid fermentation in which one amino acid acts as an electron donor and the other as an electron receptor, whereas certain amino acids, such as leucine, can act as both electron receptor and electron donor for amino acid catabolism (Prival and Magasanik, 1971; Sharma and Melkania., 2018). In this study, *K. aerogenes* was present only in the *S. chuatsi* groups feeding on the compound diet (1.70%), and its relative abundance was significantly higher in group Fl (3.30%) than that in group Fs (0.09%). We also found *E. coli* in group Fl, with a relative abundance of 0.21%. Consequently, we speculated that *K. aerogenes* may interact with *E. coli* in *S. chuatsi* to promote their ability to utilize amino acids. The combined feed is rich in protein (＞50%); it is therefore rational to assume that *K. aerogenes* may activate proline oxidase to better utilize proline and thus promote the growth of *S. chuatsi* feeding on the compound diet. In addition, *Woeseia* was detected in group Fl; it was first isolated from coastal sediment in 2016 (Du et al., 2016) and has been found mainly reproducing in seas and estuaries (Zhang et al., 2020; Guo et al., 2021). However, there are no reports on the presence of *Woeseia* in fish guts. Hence, the potential role of *Woeseia* in the fish gut remains unknown.

### Higher Diversity of Gut Microbiota in *S. chuatsi* Fed on Compound Diets

The diversity of fish gut microbiota is associated with the external environment, growth and developmental stages, nutritional status, as well as the complexity of the reproductive and digestive systems (Kim et al., 2007; Nayak, 2010; Stephens et al., 2016). Analysis of the α-diversity indicators (vital community structure parameters of the intestinal microbiota) revealed that the intestinal microbiota of *S. chuatsi* fed with compound feeds (Fl and Fs groups) were significantly higher than that of group B, whereas there was no significant difference in the diversity index of the intestinal microbiota between group Fl and group Fs, similar to *Symphysodon haraldi* and *Oncorhynchus mykiss* with different growth rates (Chapagain et al., 2019; Zhang et al., 2021). It is interesting to note that *S. chuatsi* hybrids (*S. chuatsi* ♀ × *S. scherzeri* ♂) (44.6 ± 5.4 g) fed with *Hypophthalmicthys molitrix* for 10 weeks had a significantly richer diversity of intestinal microbiota than that of the group fed with compound feeds (Li et al., 2017). These contrasting results indicate that bait and formulate feed have different effects on the intestinal microbial diversity of different fish, which may be related to fish species, size, and duration of culture cycle, or the analysis method used for determining the microbial population.

### Relationship Between the Intestinal Microbiota Compositions and Functions

We used Tax4Fun to predict the pathways related to the feed acceptance and growth of *S. chuatsi* and found that the gut microbial communities of *S. chuatsi* in different groups exhibited varied functional diversities. The OTUs involved in metabolism were the most prevalent in the gut microbes, which is similar to that found in turbot and Pacific white shrimp (Gao et al., 2018). KEGG pathway analysis revealed that the relative abundance of OTUs involved in carbohydrate metabolism and enzyme activity was higher than group Fl, indicating that the growth performance of *S. chuatsi* in this group was directly influenced by the carbohydrate metabolism capacity and balance of the intestinal environment. However, the relationship between the metabolic regulation of intestinal microbiota and changes in *S. chuatsi* requires further investigation.

## Conclusion

To summarize, this is the first analysis of gut microbial communities of *S. chuatsi* cultured for months-long. High-throughput sequencing technology was used to analyze the intestinal microbiota in *S. chuatsi* groups fed with either a compound diet (including group Fl and group Fs) or live bait for months-long. The diversity and composition of the gut microorganisms differed significantly between the compound feed and live bait groups. Furthermore, some intestinal microbiota were commonly present, or present with a relatively higher content in *S. chuatsi* that were fed artificial feed, including *Lactococcus* and *Klebsiella*, suggesting that they are closely related to the acceptance, digestion, and absorption of feedstuff. The higher relative abundance of the OTUs in group Fl are mainly involved in the metabolism of cofactors and vitamins, enzyme families and energy metabolism. Our results will be helpful for the development and application of compound feeds for *S. chuatsi*.

## Data Availability

The data presented in the study are deposited in the SRA repository, accession number PRJNA795654.
