# Supplementary material for: Comparative Analysis of the Gut Microbiota of Mandarin Fish (Siniperca chuatsi) Feeding on Compound Diets and Live Baits
Source: Front Genet. 2022 May 18;13:797420. doi: 10.3389/fgene.2022.797420 (PMC9158118; doi:10.3389/fgene.2022.797420)
Supplement: Supplementary file 7 [file DataSheet1.docx]

| Feed ingredients of the compound diet (%) | |
| --- | --- |
| Crude protein | ≥50.0 |
| Crude fat | ≥5.0 |
| Crude fibre | ≤6.0 |
| Crude ash | ≤24.0 |
| Total phosphorus | ≥1.0 |
| [Lysine](../AppData/Local/youdao/dict/Application/8.10.3.0/resultui/html/index.html#/javascript:;) | ≥2.4 |
| [Water content](../AppData/Local/youdao/dict/Application/8.10.3.0/resultui/html/index.html#/javascript:;) | ≤12.0 |

**Supplement Table 1.** Feed ingredients of the compound diet of *Siniperca chuatsi*

| Taxonomy | F | WF |  |  |  | Taxonomy | B | WB |  |  |  |
| --- | --- | --- | --- | --- | --- | --- | --- | --- | --- | --- | --- |
| *Lactococcus_garvieae* | 4.19% | 0.00% | *Lactobacillus_reuteri* | 0.08% | 0.39% | *Herbaspirillum_huttiense* | 18.42% | 0.01% | *Brevundimonas_bullata* | 0.19% | 0.01% |
| *Methylobacterium_brachiatum* | 12.39% | 0.03% | *Absiella_dolichum* | 0.10% | 0.00% | *Plesiomonas_shigelloides* | 15.32% | 0.08% | *Streptococcus_pneumoniae* | 0.02% | 0.31% |
| *Klebsiella_aerogenes* | 1.70% | 0.02% | *Lactobacillus_murinus* | 0.10% | 0.02% | *Methylobacterium_brachiatum* | 8.54% | 0.01% | *Escherichia_coli* | 0.21% | 0.00% |
| *Herbaspirillum_huttiense* | 7.66% | 0.01% | *Bacteroides_massiliensis* | 0.08% | 0.00% | *Limnohabitans_sp_103DPR2* | 0.01% | 14.95% | *Exiguobacterium_sp_ZWU0009* | 0.09% | 0.00% |
| *Escherichia_coli* | 1.36% | 0.00% | *Lolium_perenne* | 0.00% | 0.31% | *Acinetobacter_sp_CIP_53.82* | 0.30% | 0.00% | *Deinococcus_ficus* | 0.08% | 0.00% |
| *Limnohabitans_sp_103DPR2* | 0.06% | 10.05% | *Brevundimonas_bullata* | 0.28% | 0.01% | *Aeromonas_veronii* | 0.44% | 0.17% | *Nannochloropsis_gaditana* | 0.00% | 0.36% |
| *Plesiomonas_shigelloides* | 1.38% | 0.17% | *Bifidobacterium_breve* | 0.07% | 0.00% | *Moraxella_catarrhalis* | 0.17% | 0.93% | *Pararheinheimera_chironomi* | 0.07% | 0.01% |
| *Kosakonia_cowanii* | 0.70% | 0.06% | *Stenotrophomonas_maltophilia* | 0.15% | 0.00% | *Corynebacterium_accolens* | 0.20% | 0.05% | *Candidatus_Planktophila_versatilis* | 0.00% | 0.26% |
| *Bacteroides_plebeius* | 0.53% | 0.00% | *Anaerostipes_hadrus* | 0.06% | 0.00% | *Lactococcus_garvieae* | 0.27% | 0.00% | *Mycobacterium_sp* | 0.00% | 0.25% |
| *Faecalibacterium_prausnitzii* | 0.35% | 0.01% | *Serratia_marcescens* | 0.19% | 0.01% | *Populus_alba* | 0.17% | 0.01% | *Vogesella_fluminis* | 0.00% | 0.24% |
| *Bacteroides_dorei* | 0.33% | 0.00% | *Bacillus_anthracis* | 0.09% | 0.00% | *Acinetobacter_johnsonii* | 0.21% | 0.01% | *Devosia_riboflavina* | 0.08% | 0.01% |
| *Lactobacillus_johnsonii* | 0.22% | 0.34% | *Corynebacterium_propinquum* | 0.00% | 0.31% | *Flavobacterium_glycines* | 0.00% | 0.89% | *Serratia_marcescens* | 0.06% | 0.01% |
| *Candidatus_Planktophila_versatilis* | 0.00% | 1.92% | *Pediococcus_acidilactici* | 0.00% | 0.18% | *Neisseria_mucosa* | 0.01% | 0.29% | *Bacillus_anthracis* | 0.04% | 0.00% |
| *Anaerotignum_lactatifermentans* | 0.17% | 0.00% | *Acinetobacter_johnsonii* | 0.21% | 0.06% | *Corynebacterium_propinquum* | 0.14% | 0.05% | *Rhodococcus_fascians* | 0.03% | 0.00% |
| *Lactobacillus_plantarum* | 0.00% | 0.59% | *Corynebacterium_accolens* | 0.00% | 0.29% | *Staphylococcus_caprae* | 0.14% | 0.13% | *Haemophilus_parainfluenzae* | 0.01% | 0.10% |
| *Bacteroides_ovatus* | 0.12% | 0.00% | *Lactococcus_lactis* | 0.06% | 0.00% | *Lactobacillus_murinus* | 0.14% | 0.00% | *Cutibacterium_acnes* | 0.02% | 0.08% |
| *Moraxella_catarrhalis* | 0.00% | 0.87% | *Pseudomonas_balearica* | 0.08% | 0.00% | *Acinetobacter_lwoffii* | 0.24% | 0.02% | *Rothia_aeria* | 0.00% | 0.06% |
| *Aeromonas_veronii* | 0.32% | 0.77% | Others | 66.98% | 83.57% | *Delftia_tsuruhatensis* | 0.01% | 0.69% | Others | 54.37% | 80.01% |

| Taxonomy | Fl | WF |  |  |  | Taxonomy | Fs | WF |  |  |  |
| --- | --- | --- | --- | --- | --- | --- | --- | --- | --- | --- | --- |
| Lactococcus_garvieae | 8.22% | 0.00% | Pediococcus_acidilactici | 0.00% | 0.18% | Methylobacterium_brachiatum | 13.31% | 0.03% | Bacteroides_massiliensis | 0.15% | 0.00% |
| Methylobacterium_brachiatum | 11.47% | 0.03% | Corynebacterium_accolens | 0.00% | 0.29% | Herbaspirillum_huttiense | 6.19% | 0.01% | Lolium_perenne | 0.00% | 0.31% |
| Klebsiella_aerogenes | 3.30% | 0.02% | Lactococcus_lactis | 0.10% | 0.00% | Escherichia_coli | 2.51% | 0.00% | Brevundimonas_bullata | 0.33% | 0.01% |
| Herbaspirillum_huttiense | 9.12% | 0.01% | Pseudomonas_balearica | 0.11% | 0.00% | Limnohabitans_sp_103DPR2 | 0.12% | 10.05% | Bifidobacterium_breve | 0.14% | 0.00% |
| Limnohabitans_sp_103DPR2 | 0.00% | 10.05% | Escherichia_coli | 0.21% | 0.00% | Bacteroides_plebeius | 1.06% | 0.00% | Stenotrophomonas_maltophilia | 0.24% | 0.00% |
| Plesiomonas_shigelloides | 1.88% | 0.17% | Bacteroides_dorei | 0.11% | 0.00% | Faecalibacterium_prausnitzii | 0.69% | 0.01% | Anaerostipes_hadrus | 0.12% | 0.00% |
| Kosakonia_cowanii | 1.25% | 0.06% | Acinetobacter_lwoffii | 0.17% | 0.02% | Bacteroides_dorei | 0.55% | 0.00% | Serratia_marcescens | 0.14% | 0.01% |
| Candidatus_Planktophila_versatilis | 0.00% | 1.92% | Acinetobacter_johnsonii | 0.15% | 0.06% | Lactobacillus_johnsonii | 0.43% | 0.34% | Bacillus_anthracis | 0.18% | 0.00% |
| Lactobacillus_plantarum | 0.00% | 0.59% | Streptococcus_pneumoniae | 0.01% | 0.15% | Candidatus_Planktophila_versatilis | 0.00% | 1.92% | Corynebacterium_propinquum | 0.00% | 0.31% |
| Moraxella_catarrhalis | 0.00% | 0.87% | Staphylococcus_caprae | 0.00% | 0.17% | Plesiomonas_shigelloides | 0.88% | 0.17% | Kosakonia_cowanii | 0.15% | 0.06% |
| Aeromonas_veronii | 0.27% | 0.77% | Vogesella_fluminis | 0.00% | 0.24% | Anaerotignum_lactatifermentans | 0.33% | 0.00% | Pediococcus_acidilactici | 0.00% | 0.18% |
| Lactobacillus_reuteri | 0.01% | 0.39% | mixed_culture_isolate_koll13 | 0.05% | 0.00% | Lactobacillus_plantarum | 0.00% | 0.59% | Corynebacterium_accolens | 0.00% | 0.29% |
| Lactobacillus_johnsonii | 0.01% | 0.34% | Stenotrophomonas_maltophilia | 0.05% | 0.00% | Bacteroides_ovatus | 0.25% | 0.00% | Acinetobacter_johnsonii | 0.27% | 0.06% |
| Lolium_perenne | 0.00% | 0.31% | Roseomonas_ludipueritiae | 0.04% | 0.00% | Moraxella_catarrhalis | 0.00% | 0.87% | Pararheinheimera_chironomi | 0.15% | 0.01% |
| Corynebacterium_propinquum | 0.00% | 0.31% | Lactobacillus_amylovorus | 0.00% | 0.07% | Lactobacillus_reuteri | 0.15% | 0.39% | denitrifying_bacterium_enrichment_culture_clone_NOB_2_E8 | 0.08% | 0.00% |
| Serratia_marcescens | 0.24% | 0.01% | Sphingobacterium_faecium | 0.05% | 0.00% | Absiella_dolichum | 0.19% | 0.00% | Lactococcus_garvieae | 0.16% | 0.00% |
| Brevundimonas_bullata | 0.24% | 0.01% | Nitrospira_bacterium_SG8_3 | 0.03% | 0.00% | Lactobacillus_murinus | 0.20% | 0.03% | Klebsiella_aerogenes | 0.09% | 0.02% |
| Delftia_tsuruhatensis | 0.04% | 0.10% | Others | 62.86% | 82.84% | Aeromonas_veronii | 0.38% | 0.77% | Others | 70.58% | 83.56% |

Table Supplement 2. Relative abundance of microflora at the species level in the gut samples and water environment of each *S. chuatsi* group (top 35)
